# Efficacy of cinnamon in reducing anthropometric measurements in individuals with type 2 diabetes: a clinical trial

**DOI:** 10.1590/0034-7167-2024-0380

**Published:** 2025-07-11

**Authors:** José Cláudio Garcia Lira, Márcio Flávio Moura de Araújo, Jardeliny Corrêa da Penha, Maria Augusta Rocha Bezerra, Bruna Karen Cavalcante Fernandes, Thatiana Araújo Maranhão, José Wicto Pereira Borges, Marta Maria Coêlho Damasceno

**Affiliations:** I Universidade Federal do Piauí. Floriano, Piauí, Brazil; II Fundação Oswaldo Cruz. Eusébio, Ceará, Brazil; III Universidade Federal do Piauí. Picos, Piauí, Brazil; IV Universidade Estadual do Piauí. Parnaíba, Piauí, Brazil; V Universidade Federal do Piauí. Teresina, Piauí, Brazil; VI Universidade Federal do Ceará. Fortaleza, Ceará, Brazil

**Keywords:** Cinnamon, Type 2 Diabetes, Anthropometry, Clinical Trial, Overweight, Canela, Diabetes Tipo 2, Antropometría, Ensayo Clínico, Sobrepeso

## Abstract

**Objectives:**

to evaluate the effectiveness of cinnamon (*Cinnamomum verum*) in reducing anthropometric measurements in individuals with type 2 diabetes.

**Methods:**

a randomized, triple-blind, placebo-controlled clinical trial was conducted with 140 participants, divided into an experimental group (*n* = 71) and a control group (*n* = 69). The experimental group consumed 3 g/day of encapsulated cinnamon for 90 days. Anthropometric variables were measured at baseline and at the end of the study, with intra-group comparisons performed using a paired *t*-test.

**Results:**

the majority of participants were women (69.2%) with a mean age of 61 years. Reductions were observed in weight (-0.8 kg, *p* =0.11), body mass index (-0.36 kg/m^2^, *p* =0.16), body adiposity index (-1.02%, *p* =0.38), waist circumference (-3.97 cm, *p* =0.67), neck circumference (-0.45 cm, *p* = 0.68), and hip circumference (-1.83 cm, *p* =0.49), none of which were statistically significant (*p* > 0.05).

**Conclusions:**

cinnamon supplementation resulted in reductions in anthropometric measurements compared to placebo, but these differences were not statistically significant.

## RESUMEN

**Objetivos:**

analizar la eficacia de la canela (*Cinnamomum verum*) en la reducción de medidas antropométricas en personas con diabetes tipo 2.

**Métodos:**

ensayo clínico aleatorizado, controlado con placebo, triple ciego, realizado con 140 participantes divididos en un grupo experimental (n = 71) y un grupo de control (n = 69). El grupo experimental consumió 3 g/día de canela encapsulada durante 90 días. Se midieron variables antropométricas al inicio y al final del estudio, comparadas dentro de cada grupo mediante la prueba t pareada.

**Resultados:**

la mayoría de los participantes eran mujeres (69,2%), con una edad media de 61 años. Se observó una reducción en el peso (-0,8 kg, p =0,11), índice de masa corporal (-0,36 kg/m^2^, p =0,16), índice de adiposidad corporal (-1,02%, p =0,38), circunferencia de la cintura (-3,97 cm, p =0,67), circunferencia del cuello (-0,45 cm, p =0,68) y circunferencia de la cadera (-1,83 cm, p =0,49), sin significación estadística (p > 0,05).

**Conclusiones:**

el uso de la canela redujo las medidas antropométricas en comparación con el placebo, aunque sin alcanzar significación estadística.

## INTRODUCTION

The prevalence of diabetes and obesity has increased considerably worldwide. This is mainly due to a more stressful and sedentary lifestyle, diets high in fats and sugars, the failure of obesity prevention policies, and the aging process, a stage in which chronic conditions tend to become more frequent^(1,2)^.

Global estimates suggest that more than four billion people will be affected by overweight and obesity by 2035, accounting for 24% of the world’s population, including adults, children, and adolescents^(3)^. Regarding diabetes, by 2045, more than 783 million people will have the disease, with nearly 80% of these cases occurring in developing countries such as Brazil^(4)^.

Closely related, the accumulation of adiposity and insulin resistance drive microenvironmental changes that impair insulin signaling, accelerate the deterioration of pancreatic beta-cell function, and dysregulate the gut-brain microbiome axis. These disruptions lead to glycemic dysregulation, excessive production of inflammatory cytokines, and negative cardiovascular outcomes^(1,5)^.

Given these pathophysiological connections, current therapeutic options for managing diabetes and obesity share several similarities, including lifestyle interventions, pharmacotherapy, medical devices, and bariatric surgery^(6,7)^. In this context, the adjunctive use of herbal medicines, such as cinnamon, has also gained attention^(8)^.

Cinnamon and its compounds influence pathways that regulate insulin release, aiding in glycemic control and appetite modulation. For example, cinnamon can inhibit the activity of enzymes such as alpha-amylase and alpha-glucosidase, which are responsible for carbohydrate digestion, or even increase thermogenesis, contributing to weight reduction^(8)^. Moreover, clinical trials on the topic have shown promising effects of cinnamon supplementation in lowering glycemic levels and reducing body adiposity^(9-11)^.

However, evidence on the efficacy of this herbal remedy in reducing anthropometric measurements-widely used to assess overweight and obesity, as well as predictors of reduced insulin sensitivity-remains scarce. Therefore, the following hypothesis is proposed: cinnamon powder is effective in reducing anthropometric measurements in individuals with diabetes.

## OBJECTIVES

To analyze the efficacy of cinnamon (*Cinnamomum verum*) in reducing anthropometric measurements in individuals with type 2 diabetes.

## METHODS

### Ethical Aspects

This study was approved by the Research Ethics Committee for Human Subjects and registered in The Brazilian Registry of Clinical Trials (ReBEC) under identification RBR-2KKB6D, following the resolutions of the Brazilian National Health Council Nos. 466/12, 510/2016, and 580/2018.

### Study Design, Period, and Location

This is an experimental study, specifically a randomized, triple-blind, placebo-controlled clinical trial with a 1:1 allocation ratio. It was conducted between August 2019 and January 2020 with individuals diagnosed with type 2 diabetes who were registered and monitored in Primary Health Care Units (UBS in Portuguese) in a city in northeastern Brazil. The study adhered to the recommendations of the CONSORT Statement.

### Sample, Inclusion, and Exclusion Criteria

The sample size was determined based on previous studies with the same outcome^(12)^, resulting in a total of 128 participants, with 64 in the experimental group (EG) and 64 in the control group (CG). Considering potential attrition, a 20% increase was applied, requiring the recruitment of 154 participants. In the study, out of 160 individuals recruited, the final sample consisted of 100 participants, with 71 in the EG and 69 in the CG.

Eligibility criteria included men and women aged 18 to 80 years, diagnosed with type 2 diabetes, using oral antidiabetic medications, and having preserved cognitive function, as assessed by the Mini-Mental State Examination. Exclusion criteria included individuals with self-reported allergies to cinnamon, those using herbal medicines for diabetes treatment, insulin users, pregnant or breastfeeding women, and individuals diagnosed with hepatic disease, heart failure, renal failure, or respiratory disease. Discontinuation criteria included confirmed adverse events or failure to attend data collection stages.

### Study Protocol

First, the researchers determined the number of UBS to be used for data collection. This number was based on the total number of patients with type 2 diabetes (T2DM) monitored at each unit. On average, each UBS served 50 patients diagnosed with T2DM and had the minimum required infrastructure to conduct the study, including rooms for biological sample collection, patient screening, and nursing consultations, as well as morning and afternoon service hours.

The selection of UBS was conducted using a free online randomization program, where the names of all UBS were entered. After randomization, the selected UBS were visited in the defined order, and the necessity of meeting the minimum sample size was assessed. For example, if the first randomly selected UBS, “A”, included only 30 participants, the second UBS, “B”, would then be considered, and the process would continue until the required number of participants was reached.

To initiate data collection, the principal investigator visited the five UBS selected for the study, presenting the research objectives to nurses and community health agents. During these visits, printed invitations were also distributed to individuals with type 2 diabetes in each area, containing eligibility criteria and key information about the data collection process.

Data collection was conducted by a trained research team. Training included explanations about the tested product, the administration of a clinical interview for patient data collection, and the assessment of anthropometric measurements. Before data collection, the Free and Informed Consent Form was read to potential participants at the UBS where they were registered and monitored. Data collection commenced only after participants signed two copies of the consent form.

A structured questionnaire was used for data collection, including socioeconomic variables (age, sex, skin color, education, employment status, marital status, family income, cohabitation status), clinical variables (family history of diabetes, time since diagnosis, blood pressure), lifestyle-related variables (physical activity, alcohol consumption, tobacco use), and anthropometric variables (weight, height, body mass index, body adiposity index, waist circumference, hip circumference, neck circumference, and thigh circumference).

Regarding anthropometric measurements, weight was measured in kilograms (kg) using a portable digital scale (Tec-Silver Techline^©^) with a precision of 0.1 kg. Height was measured in centimeters (cm) using a non-stretchable measuring tape with a precision of 0.1 cm. Participants were instructed to stand upright and still, with their hands resting flat on their thighs and their heads aligned with the Frankfurt plane.

The body mass index (BMI) was calculated as the ratio of weight (kg) to height (m) squared (kg/m^2^). The body adiposity index (BAI) was calculated using the formula: 100% × (hip circumference) / (height × √height) - 18), with cutoff points of 25% for men and 35% for women^(13)^.

Waist circumference (WC) was measured using a non-stretchable measuring tape with 0.1 cm precision at the midpoint between the last rib and the upper edge of the iliac crest at the end of the respiratory cycle. Elevated values were defined as ≥ 90 cm in men and ≥ 80 cm in women.

Neck circumference (NC) was measured at the midpoint of the neck, between the mid-cervical spine and the anterior median region of the neck, with a precision of up to 1 mm, using a non-stretchable tape, with participants standing upright. In men, the measurement was taken just below the laryngeal prominence. NC was considered elevated when values were ≥ 39 cm in men and ≥ 35 cm in women.

To expand the findings, additional anthropometric indices were also assessed, including the waist-to-hip ratio (cutoff point: < 0.85 for women and < 0.90 for men), waist-to-thigh ratio, neck-to-thigh ratio, and waist-to-height ratio (cutoff point: 0.5)^(13)^.

The intervention in the EG consisted of administering 3 grams of encapsulated cinnamon (*Cinnamomum verum*), taken daily for 90 days. The dosage and duration were based on previous studies^(13)^. Meanwhile, the CG received placebo capsules containing microcrystalline cellulose. Both the capsules and the bottles were identical in appearance, produced by the same laboratory, differing only in numerical identification provided by the laboratory. Each capsule contained 750 mg of either cinnamon or microcrystalline cellulose, and participants in both groups were instructed to take four capsules per day: two 30 minutes before breakfast and two before lunch or dinner, accompanied by water.

Every 25 days, participants were required to visit their assigned UBS to receive a new bottle of capsules. If they failed to attend on the scheduled date and time, telephone contact was attempted, and, if necessary, a home visit was scheduled for delivery.

Randomization was performed according to UBS stratification, using 1:1 allocation in parallel groups, with six-person blocks, based on glycated hemoglobin values collected days before capsule distribution. The entire process was conducted by a research team member who had no contact with participants and did not participate in data collection.

Each participant was assigned a unique identification number, which was recorded in an Excel spreadsheet and written format, accessible only to the principal investigator at the end of the study. The study followed a triple-blind design, meaning that participants, data collectors, and statistical analysts were all blinded to group allocation.

### Analysis of Results and Statistics

Data analysis was conducted per protocol, and all variables were analyzed descriptively using numerical and visual summaries. The two groups were compared to assess differences between variables, followed by the application of the t-test for independent groups for numerical variables and the chi-square test for categorical variables. The study outcomes (weight, BMI, BAI, WC, and NC) were compared within groups using the paired t-test for dependent groups. To evaluate the percentage (%) of classification changes in participants’ markers throughout the study, contingency tables were constructed. The tests were performed using SPSS software, version 24. A 95% confidence interval was applied, and the significance level was set at p < 0.05.

## RESULTS

**Figure 1:**
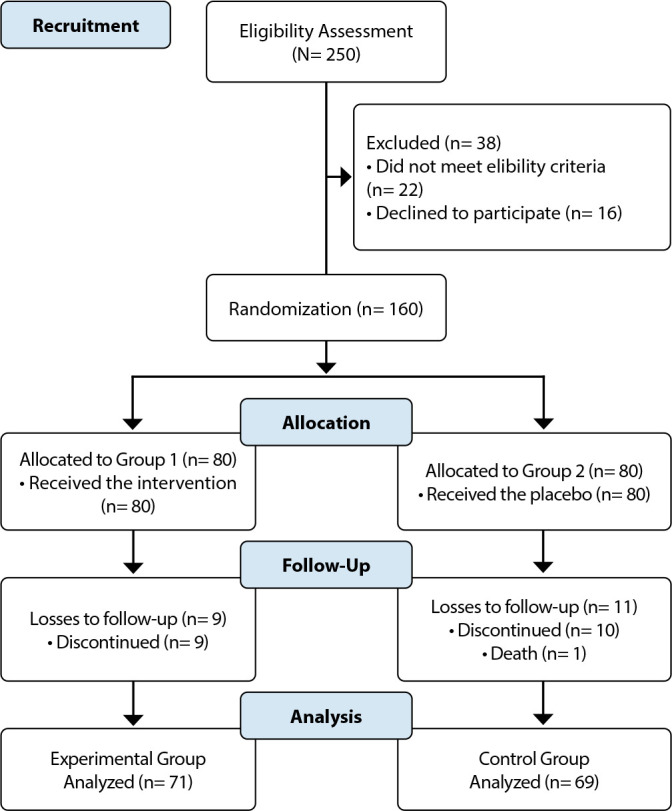
Clinical trial flowchart, Parnaíba, Piauí, Brazil, 2024

In this study, 250 individuals were recruited. However, after applying eligibility criteria and accounting for follow-up losses, the final sample consisted of 140 participants, with 71 in the EG and 69 in the CG. Homogeneity was observed between the analyzed groups (Figure 1).

Among the 140 study participants, the majority were female (n = 97, 69.2%), with a mean age of 61 years (SD = 11.7). Most identified as mixed-race (57.1%), had up to nine years of education (52.1%), were married (65.6%), and had a monthly household income of R$1,954.50. Half of the participants were retired. A family history of diabetes was reported by 64.8% (n = 46) of the EG and 71.0% (n = 49) of the CG (p = 0.27), indicating no significant differences between the groups. Regarding the duration of diabetes diagnosis, most participants in both groups had been diagnosed between 5 and 10 years (40.8% in the EG and 40.6% in the CG). Physical activity was reported by 29.6% (n = 21) of EG participants and 34.8% (n = 24) of CG participants (p = 0.32). Alcohol consumption was similar between the groups (EG = 12.7%, CG = 11.6%), as was tobacco use (EG = 5.6%, CG = 8.7%) (Table 1).

**Table 1:** Socioeconomic and clinical characteristics of participants with type 2 diabetes by allocation group, Parnaíba, Piauí, Brazil, 2024, (N = 140)

Variable	Experimental Group (n= 71)	Control Group (n= 69)	*p* value^*^
Age, mean (standard deviation)	61.7 (11.7)	60.8 (10.8)	0.66
Median (min, max)	63.0 (29. 87)	61.0 (36. 79)	
Sex, % (n)			
Female	71.8 (51)	66.7 (46)	
Male	28.2 (20)	33.3 (23)	0.58
Skin color, % (n)			
Mixed-race	56.3 (40)	58.0 (40)	1.00
White	29.6 (21)	29.0 (20)	
Black	11.3 (08)	11.6 (08)	
Asian	2.8 (02)	1.4 (01)	
Education level, % (n)			
Illiterate	11.3 (08)	13.0 (09)	0.61
Completed Primary Education	53.5 (38)	50.7 (35)	
Completed High School	23.9 (17)	30.4 (21)	
Completed Higher Education	11.3 (08)	5.8 (04)	
Employment status, % (n)			
Retired	50.7 (36)	49.3 (34)	0.26
Homemaker	25.4 (18)	17.4 (12)	
Formal/Informal Worker	22.5 (16)	26.1 (18)	
Unemployed	1.4 (01)	7.2 (05)	
Marital status, % (n)			
Married	63.7 (46)	67.5 (47)	0.58
Single	18.8 (14)	12.5 (08)	
Widowed	17.5 (11)	20.0 (14)	
Living arrangement, % (n)			
With family members	67.6 (48)	65.2 (45)	0.42
With a partner	25.4 (18)	31.9 (22)	
Alone	7.0 (05)	2.9 (02)	
Monthly family income (R$), mean (SD)	1,873 (1,327)	2,036 (1,592)	0.51
Family history of diabetes, % yes (n)	64.8 (46)	71.0 (49)	0.27
Duration of diabetes diagnosis, % yes (n)			0.68
Less than 5 years	28.2 (20)	33.3 (23)	
5 to 10 years	40.8 (29)	40.6 (28)	
11 to 20 years	19.7 (14)	20.3 (14)	
More than 20 years	11.3 (08)	5.8 (04)	
Physical exercise, % yes (n)	29.6 (21)	34.8 (24)	0.32
Alcohol consumption, % yes (n)	12.7 (09)	11.6 (08)	0.52
Tobacco use, % yes (n)	5.6 (04)	8.7 (06)	0.35

* *p value for the independent sample t-test for numerical variable means and the exact test p-value for contingency table analysis for all other variables.*

At the beginning of the study, the groups were similar in terms of anthropometric measurements, and the majority of participants in both groups were classified as overweight or obese according to BMI and BAI. The median abdominal circumference was 98 cm (range: 61 to 122 cm) in the eg and 100 cm (range: 74 to 133 cm) in the CG. Considering the cutoff point of 80-90 cm, both groups had mean values above this range, indicating a prevalence of abdominal obesity among the participants.

Likewise, the mean NC was 38 cm (SD = 3) in the eg and 37 cm (SD = 3) in the CG, with a p-value of 0.79, indicating similarity between the groups. The mean values were within the cutoff range of 35-39 cm, suggesting an increased risk for metabolic conditions. These results demonstrate that the anthropometric characteristics of the participants were comparable at the beginning of the study, with both groups showing values indicative of a risk for metabolic complications (Table 2).

**Table 2:** Measures of central tendency for anthropometric measurements at the beginning of the study in individuals with type 2 diabetes, by allocation group, Parnaíba, Piauí, Brazil, 2024, (N = 140)

Variable	Experimental Group (n= 71)	Control Group (n= 69)	*p* value^*^
Weight (kg), mean (SD)	69.0 (12. 8)	69.3 (12.8)	0.90
Median (min, max)	67.2 (36.0; 98.2)	69.0 (46.7; 117.0)	
Height (cm), mean (SD)	154 (9)	154 (7)	0.71
Median (min, max)	152 (139. 179)	153 (141. 170)	
Body mass index (BMI), mean (SD)	29.2 (4.8)	29.0 (4.5)	0.87
Median (min, max)	29.3 (18.6; 46.4)	28.6 (19.9; 42.5)	
BMI classification, % (n)			
Underweight	5.6 (4)	2.9 (20)	0.61
Normal weight	22.5 (16)	27.5 (19)	
Overweight or obese	71.8 (51)	69.6 (48)	
Central adiposity index, mean (SD)	36.3 (6.4)	35.7 (5.7)	0.57
Median (min, max)	36.4 (24.7; 52.3)	34.8 (24.8; 52.0)	
Central adiposity index classification, % (n)			
Obesity	77.5(55)	76.8 (53)	1.00
Abdominal circumference (cm), mean (SD)	99 (12)	100 (10)	0.63
Median (min, max)	98 (61. 122)	100 (74. 133)	
Waist circumference (cm), mean (SD)	97 (12)	97 (9)	0.86
Median (min, max)	97 (61. 123)	97 (80. 132)	
Neck circumference (cm), mean (SD)	38 (3)	37 (3)	0.79
Median (min, max)	37 (28. 46)	37 (31. 47)	
Hip circumference (cm), mean (SD)	103 (9)	103 (9)	0.82
Median (min, max)	102 (80. 125)	100 (89. 126)	
Thigh circumference (cm), mean (SD)	46 (5)	47 (6)	0.89
Median (min, max)	46 (33. 57)	46 (36. 65)	
Waist-to-hip ratio, mean (SD)	0. 94 (0.07)	0.95 (0.07)	0.49
Median (min, max)	0.94 (0.66; 1.09)	0.97 (0.80; 1.08)	
Waist-to-thigh ratio, mean (SD)	2.10 (0.26)	2.11 (0.26)	0.80
Median (min, max)	2.04 (1.61; 2.97)	2.11 (1.51; 2.86)	
Neck-to-thigh ratio, mean (SD)	0.82 (0.10)	0.81 (0.10)	0.80
Median (min, max)	0.80 (0.65; 1.24)	0.82 (0.55; 1.08)	
Waist-to-height ratio, mean (SD)	0.63 (0.08)	0.63 (0.06)	0.97
Median (min, max)	0.64 (0.37; 0.85)	0.62 (0.50; 0.80)	

* *p value from the independent sample t-test.*

Table 3 presents a comparison between the EG and the CG, showing the means and standard deviations (SD) of the changes in each assessed anthropometric measurement. There was a reduction in weight, BMI, BAI, abdominal circumference, NC, and hip circumference among participants in the EG compared to those in the CG. Indices such as waist-to-hip ratio and waist-to-height ratio showed no differences between groups. However, none of the evaluated variables demonstrated a statistically significant difference between the groups (all p-values > 0.05).

**Table 3:** Differences in anthropometric measurements (final minus initial) in individuals with type 2 diabetes, by allocation group, Parnaíba, Piauí, Brazil, 2024, (N = 140)

Variable	Experimental Group (n= 71)	Control Group (n= 69)	*p* value^*^
	Mean (SD)	Mean (SD)	
Weight (kg)	-0.80 (3.11)	0.03 (3.04)	0.11
Median (min, max)	-0.80 (-13.00; 9.80)	0.00 (-7.10; 19.30)	
Body Mass Index (BMI)	-0.36 (1.75)	0.02 (1.35)	0.16
Median (min, max)	-0.38 (-7.04; 6.73)	0.00 (-3.02; 8.81)	
Body Adiposity Index (BAI)	-1.02 (2.68)	-0.68 (1.97)	0.38
Median (min, max)	-0.54 (-8.17; 5.61)	0.00 (-6.36. 3.14)	
Abdominal circumference (cm)	-3.97 (5.08)	-3.61 (4.81)	0.67
Median (min, max)	-3.00 (-18.00; 19.00)	-3.00 (-22.00; 5.00)	
Waist circumference (cm)	0.39 (5.87)	1.25 (5.28)	0.36
Median (min, max)	0.00 (-18.00; 19.00)	0.00 (-19.00; 18.00)	
Neck circumference (cm)	-0.45 (1.58)	-0.33 (1.79)	0.68
Median (min, max)	0.00 (-4.00; 4.00)	0.00 (-5.00; 8.00)	
Hip circumference (cm)	-1.83 (4.75)	-1.33 (3.74)	0.49
Median (min, max)	-1.00 (-15.00; 10.00)	0.00 (-11.00; 6.00)	
Thigh circumference (cm)	1.41 (10.29)	-0.19 (2.87)	0.21
Median (min, max)	0.00 (-16.00; 69.00)	0.00 (-8.00. 7.00)	
Waist-to-hip ratio	0.02 (0.07)	0.02 (0.06)	0.77
Median (min, max)	0.00 (-0.18; 0.24)	0.02 (-0.21; 0.18)	
Waist-to-thigh ratio	-0.01 (0.25)	0.03 (0.15)	0.22
Median (min, max)	-0.02 (-1.19; 0.75)	0.02 (-0.39; 0.44)	
Neck-to-thigh ratio	-0.02 (0.09)	0.00 (0.05)	0.34
Median (min, max)	-0.02 (-0.47; 0.28)	0.00 (-0.15; 0.18)	
Waist-to-height ratio	0.00 (0.04)	0.01 (0.03)	0.38
Median (min, max)	0.00 (-0.15; 0.16)	0.00 (-0.13; 0.12)	

* *p value from the independent sample t-test.*

## DISCUSSION

When investigating whether cinnamon (*Cinnamomum verum*), at a dosage of 3 g/day for 90 days, is effective in reducing anthropometric measurements in individuals with type 2 diabetes, it was observed that participants in the EG experienced greater reductions in weight, BMI, BAI, abdominal circumference, NC, hip circumference, and waist-to-thigh and neck-to-thigh ratios compared to the CG. However, no statistically significant results were found, refuting the study’s initial hypothesis. Another similar study using lower doses demonstrated that cinnamon was effective in controlling weight gain and reducing waist and hip circumference measurements^(14)^.

Evidence regarding the benefits of cinnamon supplementation on anthropometric measures and indices remains conflicting. An umbrella review of seven meta-analyses found that cinnamon use significantly reduced body weight and BMI in individuals without diabetes when taken at doses ≥ 3 g/day. However, this herbal supplement had no effect on other adiposity markers^(15)^. Another meta-analysis on the topic showed that cinnamon can reduce BMI, body weight, and the waist-to-hip ratio^(16)^.

Cinnamon, when administered at doses ranging from 1 to 10 g/day, may be effective in reducing up to 1 kg in individuals with diabetes and/or excess weight when used for two to three months^(17)^. This finding aligns with the results of the present study. Additionally, a trial on the subject indicated that cinnamon supplementation may lead to greater weight reductions when used by women with polycystic ovary syndrome, individuals over 50 years of age, and those with a BMI ≥ 30 kg/m^2(17)^.

Upon analyzing BMI reductions among study participants, it was observed that the EG experienced a decrease of 0.38 kg/m^2^, corroborating findings from previous research^(14-17)^. However, an Iranian study designed to evaluate the effects of cinnamon on anthropometric indices found no significant reduction in BMI values^(14)^.

A study investigating the effects of cinnamon on weight control highlighted its role in regulating adipokines, which function as endocrine and paracrine hormones. The authors reported that treatment with cinnamon at a dosage of 7 mg/kg of body weight in overweight women over a 56-day period increased adiponectin levels, a hormone with protective effects and antidiabetic, anti-inflammatory, and antiatherogenic properties^(18)^. Another study, using cinnamon supplementation at 4-8 g/day for 10 weeks, demonstrated a reduction in resistin secretion, a hormone associated with insulin resistance, along with an increase in ghrelin levels in obese individuals with diabetes^(19)^, positioning cinnamon as a potentially important therapeutic option.

In the analysis of abdominal and WC reduction, which are predictors of cardiovascular risk and indicators of insulin resistance, although a decrease in these measurements was observed with daily cinnamon use, the literature is not unanimous regarding the significance of these findings^(13,17,20)^. However, it does report the efficacy of cinnamon in reducing the waist-to-hip ratio^(14,16)^, which was not observed in the present study.

A slight reduction in the waist-to-thigh and neck-to-thigh ratios was observed in the EG, although not statistically significant. A meta-analysis of cohort studies indicated that anthropometric indices are important risk markers for the development of type 2 diabetes, glycemic dysregulation, and even mortality, with the waist-to-height ratio showing the strongest association. Thus, it is evident that excessive general and central adiposity is linked to worse outcomes in individuals with type 2 diabetes. However, data involving these markers should be interpreted with caution due to geographic, age, ethnic, and follow-up heterogeneity^(21)^.

The inclusion of anthropometric markers and indices in diabetes care, as well as the consideration of Integrative and Complementary Health Practices, such as phytotherapy using cinnamon, are interesting possibilities for achieving treatment goals and promoting self-management of this condition. Furthermore, they may serve as important indicators of healthcare quality, particularly in Primary Health Care (PHC) settings^(22)^.

Even though no effectiveness of cinnamon use in reducing anthropometric measurements was found, the results of this study are valuable in clarifying the actual effects of this herbal treatment on these outcomes. Given the rigor of this triple-blind, placebo-controlled RCT, the publication of these findings has the potential to be included in systematic reviews with meta-analyses, increasing the sample power of such reviews and improving meta-analytic effects that clarify the impact of cinnamon on these outcomes. Additionally, it helps reduce publication bias stemming from negative intervention results.

### Study limitations

In this study, limitations such as the intervention duration, sample size, cinnamon dosage, and the lack of investigation into fat accumulation in specific regions, such as the liver, should be considered. Additionally, no adjustments were made for participants’ age and sex, which could be addressed in future research on this topic. When discussing the data, no studies were found that evaluated the efficacy of cinnamon in individuals with type 2 diabetes concerning anthropometric markers and indices such as thigh and NC, waist-to-thigh ratio, neck-to-thigh ratio, and waist-to-height ratio. This underscores the need for further research in this area, given the strong association between adiposity accumulation, excess weight, and negative diabetes management outcomes.

### Contributions to Nursing, Health, or Public Policy

The therapeutic management of individuals with diabetes remains a challenge for nurses worldwide. As key professionals in patient care across all levels of healthcare, nurses must be attentive to navigation practices and the implementation of advanced methodologies regarding the inclusion and emphasis on anthropometric measures and indices, as well as the consideration of adjunctive use of integrative and complementary practices such as cinnamon.

Although no statistically significant reductions were identified in our study, it is possible to suggest that the application of herbal treatments in clinical practice may be a valuable strategy in diabetes care, particularly in minimizing associated cardiometabolic risks. Furthermore, these findings may help guide public health policies and diabetes care strategies, supporting the implementation of effective risk factor screening methods and the usability of alternative treatments.

## CONCLUSIONS

The use of cinnamon at a dosage of 3 g/day for 90 days resulted in reductions in weight, BMI, BAI, abdominal circumference, NC, hip circumference, waist-to-thigh ratio, and neck-to-thigh ratio, although these reductions were not statistically significant. Studies evaluating the efficacy of cinnamon and other herbal treatments in reducing anthropometric measurements in individuals with diabetes should be widely encouraged.
