# Supplementary material for: Efficacy of cinnamon in reducing anthropometric measurements in individuals with type 2 diabetes: a clinical trial
Source: Rev Bras Enferm. 2025 Jul 11;78(3):e20240380. doi: 10.1590/0034-7167-2024-0380 (PMC12252926; doi:10.1590/0034-7167-2024-0380)
Supplement: Translation

# Translation: Eficácia da canela na redução de medidas antropométricas em pessoas com diabetes tipo 2: ensaio clínico

**DOI:** 10.1590/0034-7167-2024-0380pt

**Authors:** José Cláudio Garcia Lira, Márcio Flávio Moura de Araújo, Jardeliny Corrêa da Penha, Maria Augusta Rocha Bezerra, Bruna Karen Cavalcante Fernandes, Thatiana Araújo Maranhão, José Wicto Pereira Borges, Marta Maria Coêlho Damasceno

**Affiliations:** I Universidade Federal do Piauí. Floriano, Piauí, Brasil; II Fundação Oswaldo Cruz. Eusébio, Ceará, Brasil; III Universidade Federal do Piauí. Picos, Piauí, Brasil; IV Universidade Estadual do Piauí. Parnaíba, Piauí, Brasil; V Universidade Federal do Piauí. Teresina, Piauí, Brasil; VI Universidade Federal do Ceará. Fortaleza, Ceará, Brasil

**Keywords:** Canela, Diabetes Tipo 2, Antropometria, Ensaio Clínico, Sobrepeso.

## Abstract

**Objetivos:**

analisar a eficácia da canela (*Cinnamomum verum*) na redução de medidas antropométricas em pessoas com diabetes tipo 2.

**Métodos:**

ensaio clínico randomizado, controlado por placebo, triplo cego, realizado com 140 participantes divididos em grupo experimental (*n* = 71) e controle (*n* = 69). O grupo experimental ingeriu 3 g/dia de canela encapsulada por 90 dias. Variáveis antropométricas foram medidas no início e ao final do estudo, comparadas intragrupo pelo teste *t* pareado.

**Resultados:**

a maioria dos participantes era composta por mulheres (69,2%), com média de 61 anos. Observou-se redução no peso (-0,8 kg, *p* =0,11), índice de massa corporal (-0,36 kg/m^2^, *p* =0,16), índice de adiposidade corporal (-1,02%, *p* =0,38), circunferência da cintura (-3,97 cm, *p* =0,67), pescoço (-0,45 cm, *p* =0,68) e quadril (-1,83 cm, *p* =0,49), sem significância estatística (*p* > 0,05).

**Conclusões:**

o uso da canela reduziu medidas antropométricas em comparação ao placebo, porém sem significância estatística.

## INTRODUÇÃO

A prevalência de diabetes e obesidade tem aumentado consideravelmente em todo o mundo. Isso se deve, em especial, a um estilo de vida mais estressante e sedentário, dietas ricas em gorduras e açúcares, ao fracasso das políticas de combate à obesidade e ao processo de envelhecimento, fase em que as condições crônicas tendem a se tornar mais frequentes^(1,2)^.

As estimativas globais sugerem que mais de quatro bilhões de pessoas serão afetadas até 2035 pelo excesso de peso e obesidade, alcançando 24% da população mundial, incluindo adultos, crianças e adolescentes^(3)^. Quanto ao diabetes, até 2045, mais de 783 milhões de pessoas terão a doença, sendo quase 80% desse total em países em desenvolvimento, como o Brasil^(4)^.

Intimamente relacionados, o acúmulo de adiposidade e a resistência insulínica impulsionam mudanças microambientais que prejudicam a sinalização da insulina, aumentam a deterioração da função das células beta-pancreáticas e desregulam o eixo do microbioma intestino-cérebro, favorecendo desregulações glicêmicas, superprodução de citocinas inflamatórias e desfechos cardiovasculares negativos^(1,5)^.

Dadas essas conexões em sua patogênese, as atuais opções terapêuticas para o controle do diabetes e da obesidade têm algumas semelhanças, incluindo intervenções no estilo de vida, farmacoterapia, dispositivos médicos e cirurgias bariátricas^(6,7)^. Nesse contexto, também tem se ampliado o uso adjuvante de fitoterápicos, como a canela^(8)^.

A canela e seus compostos possuem vias que modulam corretamente a liberação de insulina, auxiliando no controle glicêmico e do apetite. A exemplo disso, a canela pode inibir a ação de enzimas como a alfa-amilase e a alfa-glicosidase, responsáveis pela digestão dos carboidratos, ou ainda aumentar a termogênese, contribuindo para a redução ponderal^(8)^. Ademais, ensaios clínicos sobre o tema mostraram efeitos animadores do uso da canela na redução dos níveis glicêmicos e na diminuição da adiposidade corporal^(9-11)^.

Contudo, ainda são incipientes as produções que evidenciam a eficácia desse fitoterápico na diminuição de medidas antropométricas, amplamente utilizadas para demarcar o excesso de peso e a obesidade, além de serem preditores da redução da sensibilidade insulínica. Logo, levanta-se a seguinte hipótese: o uso da canela em pó é eficaz para a redução de medidas antropométricas em pessoas com diabetes.

## OBJETIVOS

Analisar a eficácia da canela (*Cinnamomum verum*) na redução de medidas antropométricas em pessoas com diabetes tipo 2.

## MÉTODOS

### Aspectos éticos

A investigação foi aprovada pelo Comitê de Ética em Pesquisa com Seres Humanos e registrada na Registro Brasileiro de Ensaios Clínicos (ReBEC) sob a identificação RBR-2KKB6D, seguindo as resoluções do Conselho Nacional de Saúde nº 466/12, nº 510/2016 e nº 580/2018.

### Desenho, período e local do estudo

Trata-se de um estudo experimental, do tipo ensaio clínico randomizado, controlado por placebo, triplo cego, aleatorizado 1:1, conduzido entre agosto de 2019 e janeiro de 2020, com pessoas diagnosticadas com diabetes tipo 2, cadastradas e acompanhadas em Unidades Básicas de Saúde (UBS) de uma cidade do Nordeste do Brasil. O estudo seguiu as recomendações do *CONSORT Statement*.

### Amostra, critérios de inclusão e exclusão

A amostra foi baseada em pesquisas anteriores com o mesmo desfecho^(12)^, resultando em um total de 128 pessoas, sendo 64 no grupo experimental (GE) e 64 no grupo controle (GC). Estimando-se perdas, adicionou-se 20% ao número inicial, sendo necessário recrutar 154 pessoas para a pesquisa. No estudo, das 160 pessoas recrutadas, a amostra final foi composta por 100 participantes, sendo 71 no GE e 69 no GC.

Os critérios de elegibilidade foram: homens ou mulheres com idade entre 18 e 80 anos, com diagnóstico de diabetes tipo 2, em uso de antidiabéticos orais e com funções cognitivas preservadas, segundo o Miniexame do Estado Mental. Foram excluídos da triagem aqueles com alergia autorrelatada à canela, que faziam uso de fitoterápicos para tratamento do diabetes, em uso de insulina, mulheres em aleitamento ou grávidas e/ou pessoas com diagnóstico de doença hepática, insuficiência cardíaca, renal ou respiratória. A comprovação de eventos adversos ou a ausência nas etapas de coleta de dados constituíram critérios de descontinuidade.

### Protocolo do estudo

Primeiramente, os pesquisadores definiram o quantitativo de UBS a serem utilizadas para a coleta dos dados. O número foi determinado com base no total de pacientes com DM2 acompanhados em cada uma delas. Em média, cada UBS atendia 50 pacientes com diagnóstico de DM2 e possuía a estrutura mínima necessária para a realização do estudo, incluindo salas destinadas à coleta de material biológico, triagem de pacientes e consultório de enfermagem, além de atendimento nos turnos da manhã e da tarde. O sorteio foi realizado por um programa online e gratuito, no qual foram inseridos os nomes de todas as UBS. Após o sorteio, as UBS foram visitadas conforme a ordem definida, e a necessidade de alcançar a amostra mínima foi avaliada. Por exemplo, se a primeira UBS sorteada fosse a UBS “A” e nela fossem incluídos apenas 30 participantes, a UBS “B”, sorteada em segundo lugar, passaria a ser considerada, e o processo seguiria até atingir o número de participantes necessário.

Para dar início à coleta de dados, o pesquisador principal visitou as cinco UBS previamente sorteadas para compor o estudo, apresentando aos enfermeiros e aos agentes comunitários de saúde os objetivos da pesquisa. Na ocasião, também foram entregues convites impressos para serem distribuídos às pessoas com diabetes tipo 2 de cada área, contendo critérios de elegibilidade e algumas informações sobre a coleta de dados.

A coleta de dados foi realizada por uma equipe treinada. O treinamento envolveu explicações sobre o produto a ser testado, a condução de uma entrevista para captura de dados clínicos dos pacientes, bem como a avaliação das medidas antropométricas. Antes do levantamento das informações, foi feita a leitura do Termo de Consentimento Livre e Esclarecido aos potenciais participantes nas UBS em que as pessoas eram cadastradas e acompanhadas. Somente após a assinatura de concordância dos pacientes, em duas vias do termo, deu-se início à coleta dos dados.

Para a coleta, utilizou-se um instrumento contendo variáveis socioeconômicas (idade, sexo, cor da pele, escolaridade, situação laboral, situação conjugal, renda familiar, com quem mora), clínicas (histórico familiar de diabetes, tempo de diagnóstico, pressão arterial), relacionadas ao estilo de vida (exercício físico, uso de álcool, uso de tabaco) e variáveis antropométricas (peso, altura, índice de massa corporal, índice de adiposidade corporal, circunferência da cintura, circunferência do quadril, circunferência do pescoço e circunferência da coxa).

Em relação às medidas antropométricas, o peso foi avaliado em quilos (kg), utilizando-se uma balança portátil digital (Tec-Silver Techline^©^), com precisão de 0,1 kg. A altura foi medida em centímetros (cm), com fita inelástica, com precisão de 0,1 cm. Para isso, os participantes da pesquisa foram orientados a se posicionar eretos e imóveis, com as mãos espalmadas sobre as coxas e com a cabeça ajustada ao plano de Frankfurt. O índice de massa corporal (IMC) foi calculado pela razão entre o peso (kg) e a altura (m) ao quadrado (kg/m^2^). O índice de adiposidade corporal (IAC) foi calculado pela fórmula: 100%(circunferência do quadril)/ (altura x √ altura) - 18), e adotados os pontos de corte de 25% para homens e 35% para mulheres^(13)^.

A circunferência da cintura (CC) foi medida com uma fita métrica inelástica, com precisão de 0,1 cm, no ponto médio entre a última costela e a borda superior da crista ilíaca, no final do movimento respiratório. Foram considerados elevados os valores ≥ 90 cm nos homens e ≥ 80 cm nas mulheres.

Por sua vez, a circunferência do pescoço (CP) foi medida na metade do pescoço, entre a coluna cervical média e a região mediana do pescoço anterior, com precisão de até 1 mm, utilizando uma fita inelástica, com os participantes em pé. Nos homens, a medida foi realizada logo abaixo da proeminência laríngea. A CP foi considerada elevada quando os homens apresentavam valores ≥ 39 cm e as mulheres, valores ≥ 35 cm.

A fim de ampliar os achados, também foram investigados alguns índices antropométricos, tais como cintura-quadril (ponto de corte para mulheres < 0,85 e para homens < 0,90), cintura-coxa, pescoço-coxa e cintura-estatura (ponto de corte de 0,5)^(13)^.

A intervenção aplicada no GE consistiu na administração de 3 gramas de canela (*Cinnamomum verum*) encapsulada, ingerida diariamente durante 90 dias. A quantidade e o tempo de uso do produto foram baseados em estudos prévios^(13)^. Enquanto isso, o GC recebeu cápsulas de placebo contendo celulose microcristalina. Tanto as cápsulas quanto os frascos foram igualmente produzidos por um mesmo laboratório, diferenciando-se apenas por uma identificação numérica fornecida pelo laboratório. Cada cápsula continha 750 mg do produto (canela ou celulose microcristalina), e os participantes de ambos os grupos foram orientados a tomar quatro cápsulas ao longo do dia: duas 30 minutos antes do café da manhã e outras duas antes do almoço ou do jantar, acompanhadas de água.

A cada 25 dias, os participantes da pesquisa deveriam comparecer às UBS onde eram acompanhados para receber um novo frasco com as cápsulas. Caso não comparecessem no dia e horário agendados, contatos telefônicos eram realizados, e uma visita domiciliar poderia ser agendada para a entrega do frasco.

A randomização do estudo foi realizada conforme a estratificação das UBS, com uma alocação de 1:1 em grupos paralelos, utilizando blocos de seis participantes, com base nos valores de hemoglobina glicada coletados dias antes da entrega das cápsulas. Todo o procedimento foi conduzido por um membro do grupo de pesquisa que não teve contato com os participantes e não participou da coleta de dados.

Cada participante recebeu um número designado, que foi registrado em planilha Excel e de forma escrita, aos quais o pesquisador principal só teve acesso ao final da pesquisa. O cegamento do estudo foi do tipo triplo, uma vez que os participantes, os responsáveis pela coleta dos dados e os responsáveis pela análise estatística não tinham informações sobre a alocação.

### Análise dos resultados e estatística

A análise dos dados foi realizada por protocolo, e todas as variáveis foram analisadas descritivamente, por meio de sumários numéricos e visuais. Os dois grupos foram comparados para verificar as diferenças entre as variáveis e, em seguida, utilizou-se o teste t para grupos independentes nas variáveis numéricas e o teste qui-quadrado nas variáveis categóricas. Os desfechos sob estudo (peso, IMC, IAC, CC e CP) foram comparados intragrupo por meio do teste t pareado para grupos dependentes. Para observar o percentual (%) de modificação na classificação dos marcadores dos participantes ao longo do estudo, elaboraram-se tabelas de contingência. Os testes foram conduzidos no software SPSS, versão 24. Utilizou-se um intervalo de confiança de 95% e um nível de significância de p < 0,05.

## RESULTADOS

Nesse estudo, foram recrutadas 250 pessoas. Todavia, após a aplicação dos critérios de elegibilidade e das perdas no seguimento, fizeram parte da amostra final 140 participantes, sendo 71 no GE e 69 no GC. Foi identificada homogeneidade nos grupos analisados (Figura 1).

**Figura 1:** Fluxograma do ensaio clínico, Parnaíba, Piauí, Brasil, 2024

Das 140 pessoas que fizeram parte do estudo, a maioria era do sexo feminino (n= 97, 69,2%), tinham uma média de idade de 61 anos (DP = 11,7), era parda (57,1%), com até nove anos de estudo (52,1%), era casada (65,6%), e vivia com uma renda familiar mensal de R$1.954,50. Metade dos participantes eram aposentados. A história familiar de diabetes foi reportada por 64,8% (n=46) dos participantes do GE e 71,0% (n=49) do GC (p=0,27), indicando que não há diferenças entre os grupos. Em relação ao tempo de diagnóstico de diabetes, a maioria dos participantes em ambos os grupos foi diagnosticada entre 5 e 10 anos (40,8% no GE e 40,6% no GC). A prática de exercício físico foi relatada por 29,6% (n=21) dos participantes do GE e 34,8% (n=24) naqueles do GC (p=0,32). O uso de álcool foi similar entre os grupos (GE = 12,7%, GC = 11,6%), assim como o uso de tabaco (GE = 5,6%, GC = 8,7%) (Tabela 1).

**Tabela 1:** Características socioeconômicas e clínicas dos participantes com diabetes tipo 2, por grupo de alocação, Parnaíba, Piauí, Brasil, 2024, (N= 140)

Variável	Grupo Experimental (n= 71)	Grupo Controle (n= 69)	Valor de *p* ^*^
Idade, média (desvio padrão)	61,7 (11,7)	60,8 (10,8)	0,66
Mediana (mínimo, máximo)	63,0 (29, 87)	61,0 (36, 79)	
Sexo, % (n)			
Feminino	71,8 (51)	66,7 (46)	
Masculino	28,2 (20)	33,3 (23)	0,58
Cor da pele, % (n)			
Parda	56,3 (40)	58,0 (40)	1,00
Branca	29,6 (21)	29,0 (20)	
Negra	11,3 (08)	11,6 (08)	
Amarela	2,8 (02)	1,4 (01)	
Escolaridade, % (n)			
Analfabeto	11,3 (08)	13,0 (09)	0,61
Ensino Fundamental completo	53,5 (38)	50,7 (35)	
Ensino Médio completo	23,9 (17)	30,4 (21)	
Ensino Superior completo	11,3 (08)	5,8 (04)	
Tipo de trabalho, % (n)			
Aposentado(a)	50,7 (36)	49,3 (34)	0,26
Do lar	25,4 (18)	17,4 (12)	
Formal/informal	22,5 (16)	26,1 (18)	
Desempregado(a)	1,4 (01)	7,2 (05)	
Situação conjugal, % (n)			
Casado(a)	63,7 (46)	67,5 (47)	0,58
Solteiro(a)	18,8 (14)	12,5 (08)	
Viúvo(a)	17,5 (11)	20,0 (14)	
Com quem mora, % (n)			
Familiares	67,6 (48)	65,2 (45)	0,42
Companheiro(a)	25,4 (18)	31,9 (22)	
Sozinho	7,0 (05)	2,9 (02)	
Renda familiar mensal (R$), média (DP)^^**^^	1.873 (1.327)	2.036 (1.592)	0,51
História familiar de diabetes, % sim (n)	64,8 (46)	71,0 (49)	0,27
Tempo de diagnóstico de diabetes, % sim (n)			0,68
Menos de 5 anos	28,2 (20)	33,3 (23)	
De 5 a 10 anos	40,8 (29)	40,6 (28)	
De 11 a 20 anos	19,7 (14)	20,3 (14)	
Mais de 20 anos	11,3 (08)	5,8 (04)	
Prática de exercício físico, % sim (n)	29,6 (21)	34,8 (24)	0,32
Usa álcool, % sim (n)	12,7 (09)	11,6 (08)	0,52
Usa tabaco, % sim (n)	5,6 (04)	8,7 (06)	0,35

* *Valor de p para teste t para amostras independentes para médias de variáveis numéricas e p-valor do teste exato para tabela de contingência para todas as outras*

No início do estudo, os grupos apresentaram-se semelhantes quanto às medidas antropométricas, e a maioria dos participantes de ambos os grupos estava com sobrepeso ou obesidade, segundo o IMC e o IAC. A mediana da circunferência abdominal foi de 98 cm (variação: 61 a 122 cm) no GE e 100 cm (variação: 74 a 133 cm) no GC. Considerando o ponto de corte de 80-90 cm, ambos os grupos apresentaram valores médios acima desse intervalo, indicando uma prevalência de obesidade abdominal entre os participantes.

Por sua vez, a circunferência média do pescoço foi de 38 cm (DV = 3) no GE e 37 cm (DV = 3) no GC, com um *p*-valor de 0,79, indicando similaridade entre os grupos, com valores médios dentro do ponto de corte de 35-39 cm, sugerindo um risco aumentado para condições metabólicas. Esses resultados demonstram que as características antropométricas dos participantes eram comparáveis no início do estudo, com ambos os grupos apresentando valores indicativos de risco para complicações metabólicas (Tabela 2).

**Tabela 2:** Medidas de tendência central das medidas antropométricas no início do estudo de pessoas com diabetes tipo 2, por grupo de alocação, Parnaíba, Piauí, Brasil, 2024, (N= 140)

Variáveis	Grupo experimental (n= 71)	Grupo controle (n= 69)	Valor de *p* ^*^
Peso em kg, média (DV)	69,0 (12, 8)	69,3 (12,8)	0,90
Mediana (mínimo, máximo)	67,2 (36,0; 98,2)	69,0 (46,7; 117,0)	
Altura em cm, média (DV)	154 (9)	154 (7)	0,71
Mediana (mínimo, máximo)	152 (139, 179)	153 (141, 170)	
Índice de massa corporal, média (DV)	29,2 (4,8)	29,0 (4,5)	0,87
Mediana (mínimo, máximo)	29,3 (18,6; 46,4)	28,6 (19,9; 42,5)	
IMC, % (n)			
Baixo peso	5,6 (4)	2,9 (20)	0,61
Peso adequado	22,5 (16)	27,5 (19)	
Sobrepeso ou obeso	71,8 (51)	69,6 (48)	
Índice adiposidade central, média (DV)	36,3 (6,4)	35,7 (5,7)	0,57
Mediana (mínimo, máximo)	36,4 (24,7; 52,3)	34,8 (24,8; 52,0)	
Índice de adiposidade central			
Obesidade, % (n)	77.5(55)	76.8 (53)	1,00
Circunferência abdominal, média (DV)	99 (12)	100 (10)	0,63
Mediana (mínimo, máximo)	98 (61, 122)	100 (74, 133)	
Circunferência da cintura, média (DV)	97 (12)	97 (9)	0,86
Mediana (mínimo, máximo)	97 (61, 123)	97 (80, 132)	
Circunferência do pescoço, média (DV)	38 (3)	37 (3)	0,79
Mediana (mínimo, máximo)	37 (28, 46)	37 (31, 47)	
Circunferência do quadril, média (DV)	103 (9)	103 (9)	0,82
Mediana (mínimo, máximo)	102 (80, 125)	100 (89, 126)	
Circunferência da coxa, média (DV)	46 (5)	47 (6)	0,89
Mediana (mínimo, máximo)	46 (33, 57)	46 (36, 65)	
Relação cintura-quadril, média (DV)	0, 94 (0,07)	0,95 (0,07)	0,49
Mediana (mínimo, máximo)	0,94 (0,66; 1,09)	0,97 (0,80; 1,08)	
Relação cintura-coxa, média (DV)	2,10 (0,26)	2,11 (0,26)	0,80
Mediana (mínimo, máximo)	2,04 (1,61; 2,97)	2,11 (1,51; 2,86)	
Relação pescoço-coxa, média (DV)	0,82 (0,10)	0,81 (0,10)	0,80
Mediana (mínimo, máximo)	0,80 (0,65; 1,24)	0,82 (0,55; 1,08)	
Relação cintura-estatura, média (DV)	0,63 (0,08)	0,63 (0,06)	0,97
Mediana (mínimo, máximo)	0,64 (0,37; 0,85)	0,62 (0,50; 0,80)	

* *Valor de p do teste t para amostras independentes.*

A Tabela 3 apresenta uma comparação entre o GE e o GC, com as médias e desvios-padrão (DP) das mudanças em cada medida antropométrica avaliada. Houve redução no peso, no IMC, no IAC, na circunferência abdominal, na CP e na circunferência do quadril dos participantes do GE, em comparação com aqueles alocados no GC. Índices como cintura-quadril e cintura-estatura não apresentaram diferenças entre os grupos. Contudo, nenhuma das variáveis avaliadas apresentou uma diferença estatisticamente significativa entre os grupos (todos os *p*-valores > 0,05).

**Tabela 3:** Diferenças das medidas antropométricas (final menos inicial) de pessoas com diabetes tipo 2, por grupo de alocação, Parnaíba, Piauí, Brasil, 2024, (N= 140)

Variáveis	Grupo Experimental (n= 71)	Grupo Controle (n= 69)	Valor de *p* ^*^
	média (DP)	média (DP)	
Peso	-0,80 (3,11)	0,03 (3,04)	0,11
Mediana (mínimo, máximo)	-0,80 (-13,00; 9,80)	0,00 (-7,10; 19,30)	
IMC	-0,36 (1,75)	0,02 (1,35)	0,16
Mediana (mínimo, máximo)	-0,38 (-7,04; 6,73)	0,00 (-3,02; 8,81)	
IAC	-1,02 (2,68)	-0,68 (1,97)	0,38
Mediana (mínimo, máximo)	-0,54 (-8,17; 5,61)	0,00 (-6,36, 3,14)	
Circunferência abdominal	-3,97 (5,08)	-3,61 (4,81)	0,67
Mediana (mínimo, máximo)	-3,00 (-18,00; 19,00)	-3,00 (-22,00; 5,00)	
Circunferência da cintura	0,39 (5,87)	1,25 (5,28)	0,36
Mediana (mínimo, máximo)	0,00 (-18,00; 19,00)	0,00 (-19,00; 18,00)	
Circunferência do pescoço	-0,45 (1,58)	-0,33 (1,79)	0,68
Mediana (mínimo, máximo)	0,00 (-4,00; 4,00)	0,00 (-5,00; 8,00)	
Circunferência do quadril	-1,83 (4,75)	-1,33 (3,74)	0,49
Mediana (mínimo, máximo)	-1,00 (-15,00; 10,00)	0,00 (-11,00; 6,00)	
Circunferência da coxa	1,41 (10,29)	-0,19 (2,87)	0,21
Mediana (mínimo, máximo)	0,00 (-16,00; 69,00)	0,00 (-8,00, 7,00)	
Relação cintura-quadril	0,02 (0,07)	0,02 (0,06)	0,77
Mediana (mínimo, máximo)	0,00 (-0,18; 0,24)	0,02 (-0,21; 0,18)	
Relação cintura-coxa	-0,01 (0,25)	0,03 (0,15)	0,22
Mediana (mínimo, máximo)	-0,02 (-1,19; 0,75)	0,02 (-0,39; 0,44)	
Relação pescoço-coxa	-0,02 (0,09)	0,00 (0,05)	0,34
Mediana (mínimo, máximo)	-0,02 (-0,47; 0,28)	0,00 (-0,15; 0,18)	
Relação cintura-estatura	0,00 (0,04)	0,01 (0,03)	0,38
Mediana (mínimo, máximo)	0,00 (-0,15; 0,16)	0,00 (-0,13; 0,12)	

* *Valor de p do teste t para amostras independentes.*

## DISCUSSÃO

Ao se investigar se a canela (*Cinnamomum verum*), em doses de 3 g/dia, durante 90 dias, é eficaz para reduzir as medidas antropométricas de pessoas com diabetes tipo 2, identificou-se que os participantes do GE tiveram maiores reduções no peso, IMC, IAC, nas circunferências abdominal, do pescoço e do quadril, e nas relações cintura-coxa e pescoço-coxa, em comparação com o GC, ainda que não tenham sido encontrados resultados significativos, refutando a hipótese levantada pelo estudo. Outro estudo semelhante, com doses menores, mostrou que a canela foi capaz de controlar o ganho de peso e reduzir as medidas antropométricas da CC e do CD^(14)^.

Evidências sobre os benefícios da suplementação de canela nas medidas e índices antropométricos ainda são conflitantes. Uma revisão guarda-chuva de sete metanálises mostrou que o uso da canela reduziu significativamente o peso corporal e o IMC de pessoas sem diabetes, em doses ≥ 3 g/dia. Todavia, o uso desse fitoterápico não teve efeito sobre outros marcadores de adiposidade^(15)^. Outra metanálise sobre o tema mostrou que a canela é capaz de reduzir o IMC, o peso corporal e a relação cintura-quadril^(16)^.

A canela administrada em doses de 1-10 g/dia pode ser eficaz na redução de até 1 kg em pessoas com diabetes e/ou excesso de peso, quando utilizada por dois a três meses^(17)^. Isso foi semelhante ao que se identificou na presente investigação. Adicionalmente, um ensaio sobre o tema mostrou que a canela, quando utilizada por mulheres com síndrome dos ovários policísticos, pessoas com idade superior a 50 anos e com IMC ≥ 30 kg/m^2^, parece resultar em melhores reduções ponderais^(17)^.

Ao se analisar a diminuição obtida no IMC dos participantes do estudo, percebeu-se que, no GE, houve uma queda de 0,38 kg/m^2^, corroborando os achados de outras pesquisas^(14-17)^. Entretanto, um estudo iraniano desenhado para avaliar os efeitos da canela nos índices antropométricos não encontrou queda nos valores de IMC^(14)^.

Um estudo que investigou os efeitos da canela no controle ponderal ressaltou a ação desse produto na regulação de adipocinas, que atuam como hormônios endócrinos e parácrinos. Os autores constataram que um tratamento com canela na dose de 7 mg/kg de peso em mulheres com excesso de peso, durante 56 dias, aumentou os níveis de adiponectina, que desempenha um papel protetor, com propriedades antidiabéticas, anti-inflamatórias e antiaterogênicas^(18)^. Outro estudo, desta vez com suplementação de 4-8 g/dia de canela por 10 semanas, mostrou que houve redução da secreção de resistina, que está associada ao desenvolvimento de resistência insulínica, além do aumento dos níveis de grelina entre pessoas obesas com diabetes^(19)^, despontando-a como uma possibilidade terapêutica importante.

Na análise da redução da circunferência abdominal e da cintura, preditores de risco cardiovascular e indicadores de resistência insulínica, ainda que se observe diminuição dos valores dessas medidas com o uso diário da canela, a literatura não é unânime quanto à significância desses achados^(13,17,20)^, mas reporta a eficácia da canela na diminuição da relação cintura-quadril^(14,16)^, o que não foi apresentado no estudo em tela.

Por sua vez, uma discreta redução das relações cintura-coxa e pescoço-coxa foi observada no GE, embora não significativa. Uma metanálise de estudos de coorte mostrou que os índices antropométricos são importantes marcadores de risco para o desenvolvimento de diabetes tipo 2, desregulações glicêmicas e até mesmo mortalidade, sendo a relação cintura-altura aquela que possui uma associação mais forte. Dessa forma, fica evidente que o acúmulo excessivo de adiposidade geral e central está associado a piores desfechos em pessoas com diabetes tipo 2. Contudo, os dados que envolvem esses marcadores devem ser interpretados com cautela, dada a heterogeneidade geográfica, etária, étnica e de acompanhamento^(21)^.

A inclusão de marcadores e índices antropométricos nos cuidados às pessoas com diabetes e a consideração sobre o uso de Práticas Integrativas e Complementares em Saúde, como a fitoterapia, a partir do uso da canela, são possibilidades interessantes para o alcance de metas e autogestão da enfermidade em destaque. Além disso, podem configurar-se como importantes balizadores da qualidade da assistência à saúde em locais como a Atenção Primária à Saúde^(22)^.

Mesmo não se encontrando efetividade no uso da canela para a redução de medidas antropométricas, os resultados deste estudo são úteis para esclarecer os reais efeitos desse fitoterápico nesses desfechos. Considerando o rigor deste ECR, triplo cego e placebo controlado, a publicação desses resultados tem potencial para ser incluída em revisões sistemáticas com metanálises, aumentando o poder amostral dessas revisões e melhorando os efeitos metanalíticos que esclarecem os impactos da canela nesses desfechos. Além disso, diminui o viés de publicação oriundo de resultados de intervenções negativas.

### Limitações do estudo

Neste estudo, limitações como o tempo de intervenção, o tamanho da amostra, a dose da canela e a falta de investigação quanto ao acúmulo de gordura em regiões específicas, como o fígado, devem ser consideradas. Também não foram realizados ajustes quanto à idade e ao sexo dos participantes, o que pode ser incluído em pesquisas futuras sobre o tema. Para a discussão dos dados, não foram encontrados estudos que avaliaram a eficácia da canela em pessoas com diabetes tipo 2 sobre marcadores e índices antropométricos, como a circunferência da coxa e do pescoço e as relações cintura-coxa, pescoço-coxa e cintura-estatura. Isso reforça a necessidade de ampliação de investigações como esta, visto o forte entrelaçamento entre o acúmulo de adiposidade, o excesso de peso e desfechos negativos no gerenciamento do diabetes.

### Contribuições para a área da Enfermagem, Saúde ou Política Pública

A gestão terapêutica de pessoas com diabetes ainda é um desafio para enfermeiros em todo o mundo. Como protagonistas nos cuidados em todos os níveis de atenção, os enfermeiros devem estar atentos às práticas de navegação e à condução de práticas avançadas relacionadas à inclusão e ao destaque das medidas e índices antropométricos, bem como à consideração do uso adjuvante de práticas integrativas e complementares, como a canela.

Ainda que em nosso estudo não tenham sido identificadas reduções estatisticamente significativas, é possível sinalizar que a aplicabilidade de fitoterápicos na prática clínica pode ser uma estratégia perspicaz no cuidado ao paciente com diabetes, especialmente para minimizar riscos cardiometabólicos associados. Além disso, essas evidências podem orientar políticas públicas de saúde e a linha de cuidados em diabetes, auxiliando na implementação de estratégias assertivas para o rastreamento de fatores de risco e na usabilidade de tratamentos alternativos.

## CONCLUSÕES

O uso da canela em doses de 3 g/dia, durante 90 dias, apresentou reduções no peso, IMC, IAC, circunferência abdominal, CP, circunferência do quadril, relação cintura-coxa e relação pescoço-coxa, ainda que não significativas. Pesquisas que avaliem a eficácia do uso da canela e de outros fitoterápicos na redução de medidas antropométricas em pessoas com diabetes devem ser amplamente incentivadas.
